# Complete Genome Sequence of a Novel *Azospirillum* Strain TA Isolated from Western Siberia Chernevaya Taiga Soil

**DOI:** 10.3390/microorganisms12122599

**Published:** 2024-12-16

**Authors:** Mikhail Rayko, Irina Kravchenko, Alla Lapidus

**Affiliations:** 1 Laboratory of Cytology of Unicellular Organisms, Institute of Cytology RAS, 194064 St. Petersburg, Russia; mike.rayko@gmail.com; 2 Winogradsky Institute of Microbiology, Research Center of Biotechnology, Russian Academy of Sciences, 119071 Moscow, Russia; irinakravchenko@inbox.ru; 3 Independent Researcher, 125493 Moscow, Russia

**Keywords:** *Azospirillum*, PGPB, chromids, whole-genome assembly

## Abstract

A whole genome sequence of a new strain of the nitrogen-fixing bacterium *Azospirillum doebereinerae,* known for its diverse plant growth-promoting bacteria (PGPB), was obtained for the first time. The strain, designated *Azospirillum doebereinera*e AT, was isolated during a soil analysis in the Chernevaya taiga of Western Siberia, a unique and fertile forest ecosystem known for its diverse plant growth-promoting bacteria (PGPB). The *A. doebereinerae* genome under study is fully assembled into seven circular molecules, none of which are unequivocally plasmids, with a total length of 6.94 Mb and a G + C content of 68.66%. A detailed phylogenomic analysis confirmed its placement within the genus *Azospirillum*, specifically closely related to *A. doebereinerae* GSF71^T^. Functional annotation revealed genes involved in nitrogen metabolism, highlighting the potential of strain TA as a biofertilizer and plant growth-promoting agent. The findings contribute to our understanding of the genomic diversity and metabolic potential of the *Azospirillum* genus, and they are of interest for further study in the field of comparative bacterial genomics, given the strain’s multi-chromosomal genome structure.

## 1. Introduction

The genus *Azospirillum*, included in the *Rhodospirillaceae* family of the class Alphaproteobacteria, is one of the best-studied genera of plant growth promoting-bacteria (PGPB), recognized as biofertilizers because of their ability to promote plant growth and productivity [1]. Bacteria of the genus *Azospirillum* can form associations with the roots of cereals, grasses, and tuberous plants, enriching the soil with nitrogen and reducing the need for nitrogen fertilizers. According to research from throughout the world, *Azospirillum* can increase the production of agriculturally important crops in a variety of soil types and climate zones [2,3].

Following its original description, multiple plant-associated *Azospirillum* species have been shown to perform direct nitrogen fixation [4], phosphate solubilization [5], drought and salt stress alleviation [6], and root development promotion [7], among other processes [8]. Genes associated with these features have been identified in several publicly available *Azospirillum* genomes, such as *nif* (nitrogen fixation), *acdS* (ACC deaminase) [9], *pqq* (PQQ synthesis) [10], and indole acetic acid biosynthesis (e.g., *iaaH*, *iaaM* and *ipdC*) [11]. To date, there are twenty-five validly recognized *Azospirillum* species, following *A. irakense* and *A. amazonense* reclassification [12]. Recent analyses of 16S rRNA and *rpo*D gene sequences confirmed that the genus *Azospirillum* can be subdivided into two groups—Clade B (*brasilense*) and Clade L (*lipoferum*) [13].

Many *Azospirillum* species are characterized by the presence of several “megareplicons” [14], with the number and size varying even among closely related species. This feature is quite uncommon and particularly abundant in bacteria that inhabit extreme environments (such as soil), contributing to the complexity of the genome, supporting specific adaptations required for survival in unique niches [15].

In our previous work [16], we studied the bacterial and fungal composition of soils of the Chernevaya taiga, a unique forest ecosystem in Western Siberia, characterized by fertile soils, exceptionally large herbaceous plant sizes, and extraordinarily rapid rates of plant residue degradation. In that study, we demonstrated the presence of various plant growth-promoting bacteria (PGPB) using amplicon sequencing data. PGPB are beneficial microorganisms that enhance the growth and health of plants through various mechanisms, such as promoting nutrient uptake, producing growth-promoting hormones, suppressing plant pathogens, and improving tolerance to abiotic stress factors like drought or salinity. Overall, PGPB play a vital role in sustainable agricultural practices by reducing the need for chemical inputs and enhancing crop productivity.

We also studied the rhizosphere microbiome of agricultural plants grown in laboratory experiments on virgin Umbrisol from the Chernevaya taiga. The analysis of 16S S rRNA sequencing data in this study showed that the microbiome was enriched with different PGPB [17]. The culture TA attracted our attention after we isolated numerous cultivable rhizosphere PGPB. After identifying it as an *Azospirillum* strain, we sequenced its genome to investigate its metabolic capabilities, determine whether it was a distinct species, and delve into its metabolic capabilities.

In this paper, we report the results of whole-genome sequencing and comparative genomic analysis of the *Azospirillum* sp. TA strain isolated from the wheat rhizosphere during these experiments, with the aim of identifying the genes involved in the plant growth-promoting properties of this isolate. Using two sequencing technologies (Oxford Nanopore and Illumina), a complete assembly of all amplicons constituting the genome of the *Azospirillum* sp. TA strain was obtained.

## 2. Materials and Methods

### 2.1. Sample Source

Field studies were conducted in May 2020 in the Tomsk region, Russia. The studied culture was isolated from samples of dark gray soil (Retisols, FAO classification) under a tallgrass fir–aspen forest, with a shrinking fir stand, in the Chernevaya taiga (56.30693 N, 85.47063 E). On average, the annual temperature of the air varies between +1 °C and −1 °C (30–34 °F). The growing season lasts for about 90 days. The annual precipitation rate ranges from 800 to 1200 mm 153 (32–47 in.), with the largest amount of precipitation falling in the summer and a very thick snow coverage of up to 2 or 3 m remaining in place for six months at a time in the winter. The lowest temperatures in January can reach −50 °C (−60 °F), but they usually do not last for more than two weeks. The average air temperature in January is about −20 °C (−4 °F). The soil, however, remains unfrozen throughout the whole winter, protected by the thick cover of snow. A detailed description of the areas and research sites is given in a previous publication [16]. *Azospirillum* strain TA was isolated from the rhizosphere soil of wheat plants growing in the Chernevaya taiga in laboratory experiments [17].

### 2.2. Isolation Procedure

The enrichment and isolation of the nitrogen-fixing bacterium TA was performed on a semi-solid NFb growth medium, and its composition was as follows (g L^−1^): 1, sodium malate; 0.5, K_2_HPO_4_; 0.2, MgSO_4_; 0.1, NaCl; 0.02, CaCl_2_ 2H_2_O; 2 mL L_−1_ micronutrient solution containing (g L−1) 0.04, CuSO_4_ ·5H_2_O; 0.12, ZnSO_4_ ·7H_2_O; 1.40, H_3_BO_3_; 1.0, Na_2_MoO_4_ ·2H_2_O, and 1.175 MnSO_4_; and 1 mL L_−1_ vitamin solution (10 mg biotin and 20 mg pyridoxal-HCl in 100 mL distilled water). The pH was adjusted to 6.5, and the medium was sterilized at 121 °C for 15 min.

Serial ten-fold dilutions of the rhizosphere soil (0.1 mL) were inoculated into 15 mL flasks with 5 mL N-free NFb medium and incubated at 25 °C [18]. After 5–7 days, a dense film on the upper part of the medium layer was observed, and *Azospirillum*-like bacteria were identified by phase-contrast microscopy. Colonies were obtained on NFb agar (1.5%). After 10 days of incubation at 25 °C, the highest dilution showed the presence of light beige spherical colonies about 1–2 mm in diameter. Individual colonies were transferred to the same fresh media and to the medium with the addition of Congo red dye, and, after 5 passages, strain TA was obtained. The purity of the strain was confirmed by routine microscopic examination and 16S rRNA gene sequencing.

*Azospirillum* TA was routinely cultured in LB medium containing (L^−1^) 5 g peptone, 3 g beef extract, and 5 g NaCl at 30 °C. In order to characterize the isolated strain, the 16S rDNA gene was amplified by polymerase chain reaction (PCR), sequenced, and deposited in the NCBI GenBank (accession ON103335.1).

### 2.3. DNA Extraction and Sequencing

For genome sequencing, total genomic DNA was extracted from 10 mL overnight cultures using a DNA extraction kit (Power Soil DNA Isolation Kit, Qiagen, Calsbad, CA, USA) following the manufacturer’s instructions. Quantification and quality control of the genomic DNA were completed using a Qubit fluorometer (Invitrogen, Waltham, MA, USA) with a Qubit dsDNA BR Assay kit (Invitrogen, Waltham, MA, USA) and 0.7% agarose gel electrophoresis with λ-Hind III digest DNA marker (Promega, Madison, WI, USA).

Genomic DNA was sequenced on Illumina (Illumina Inc., San Diego, CA, USA) and Nanopore MinION (Oxford Nanopore, Cambridge, UK) devices. The combination of long-read Nanopore and short-read Illumina sequencing ensured high accuracy and comprehensive coverage of the genome.

### 2.4. Genome Assembly, Annotation, and Analysis

Nanopore sequencing data were assembled de novo using Flye v. 2.9.3 [19], which is optimized for long-read assemblies and generates highly contiguous genome assemblies by reconstructing contigs with overlapping sequences. The resulting draft assembly was polished with Illumina short reads using Pilon v. 1.23 [20], which corrects errors in base calls, insertions, and deletions, thereby improving the overall accuracy of the assembly. Multiple iterations of polishing were performed to ensure the highest possible quality and minimize assembly errors.

Genome annotation was performed using the Prokka pipeline v. 1.14.6 [21], a widely used tool for rapid and comprehensive bacterial genome annotation. Prokka identified and annotated coding sequences (CDSs), ribosomal RNAs (rRNAs), transfer RNAs (tRNAs), and other functional elements, using curated databases such as UniProt and RefSeq for accurate functional prediction. Default parameters were used for annotation, with adjustments made for organism-specific features where necessary, ensuring the precise annotation of genomic elements.

The complete genome assembly was deposited in the NCBI GenBank (BioProject PRJNA1182660).

### 2.5. Phylogenomic Analysis

A phylogenomic analysis was conducted to determine the phylogenetic position of the *A. doebereinerae* TA strain relative to other species in the genus. First, a set of single-copy ortholog genes was identified using BUSCO v5 [22] with the bacteria_odb10 dataset, which is specifically designed for bacterial genomes and includes 124 bacterial ortholog groups. The genome assembly was assessed for completeness, and orthologous gene sequences were extracted from the BUSCO output.

The protein sequences of these orthologs were aligned using MAFFT v7.475 [23], a multiple-sequence alignment tool optimized for speed and accuracy. The --auto mode was employed to select the most appropriate alignment algorithm based on the data size and composition, ensuring high-quality alignments.

Phylogenetic tree reconstruction was performed with IQ-TREE v2.2.0 [24], using the maximum-likelihood (ML) approach. The best-fit evolutionary model for the alignment was determined automatically by IQ-TREE, employing the ModelFinder feature. The phylogenetic tree was then generated with 1000 ultrafast bootstrap replicates to ensure the statistical robustness of the inferred relationships.

## 3. Results

### 3.1. Organism Taxonomy Characteristics

**Figure 1:**
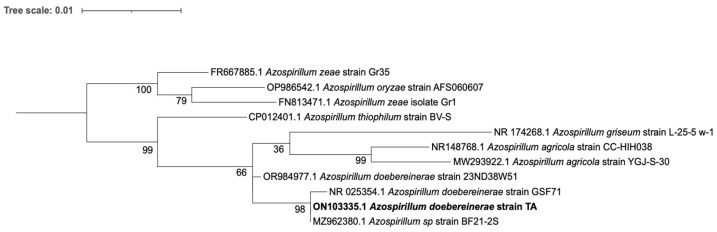
Phylogenetic tree highlighting the position of strain TA relative to other type strains within the genus *Azospirillum*. The strains are shown along with the corresponding GenBank accession numbers of their 16S rRNA genes. The sequences were aligned using MAFFT, and the maximum-likelihood tree was constructed based on the Tamura–Nei model using IQ-TREE 2.

The 16S rRNA sequence analysis revealed that the TA strain belongs to the *Azospirillum* genus, clustering with other species. *A. doebereinerae* GSF71^T^ (NR_025354.1) had the highest 16S rDNA sequence similarity with the TA strain 99.77, but the query cover was 92%. The species *A. thiophilum* BV-S^T^ (NR_116410.1), *A. griseum* L-25-5w-1^T^ (NR_174268.1), and *A. agricola* CC-HIHO38^T^ (NR_148768.1) had 100% query coverage and 97.87, 97.71, and 97.44% nucleotide identity, respectively. We carefully selected the alignment and tree inference methods to ensure that they were the most appropriate for our data, considering the characteristics of the sequences and the objectives of the analysis. MAFFT is a widely recognized tool for accurate multiple-sequence alignment, and, for phylogenetic reconstruction, the Tamura–Nei model was determined to be the most suitable for this dataset through an automated model selection process. Thus, we could describe our culture as a new strain of *A. doebereinerae* (Figure 1).

### 3.2. Assembly Description and Genome Architecture

The genome was assembled into seven circular contigs, each corresponding to one replicon (chromosome, or “chromid”). The total genome length was 6.937 Mb, with an average G + C content of 68.66%. The predicted number of protein-coding sequences (CDS) was 6248, and the number of predicted RNAs was 109 (85 tRNAs and 24 rRNAs). The detailed distribution of the genome features across the replicons is shown in Table 1.

### 3.3. Phylogenomic Analysis

Bacteria of the genus *Azospirillum* are champions in the number of large replicons in the genome (from 6 to 10) [14], and the availability of complete genomes assembled into circular contigs (corresponding to real replicons) allows us to study the evolution of replicons, their stability, and the flow of genes between them.

In *Azospirillum* spp. genomes, we found a rather rare phenomenon of rRNA genes being present on additional chromosomes (see Table 1). In such cases, potential horizontal gene transfer can distort the true topology of the phylogenetic tree. To offset this effect and provide data for further studies of the comparative genomics of the genus *Azospirillum*, we performed a phylogenomic analysis (Table 2). We analyzed all available *Azospirillum* genomes assembled into complete chromosome-level. It turned out that we had thus obtained the first complete assembly of the genome of this species—at the moment GenBank contains only two genomes of A. doebereinerae, both assembled to the contig-level only.

**Figure 2:**
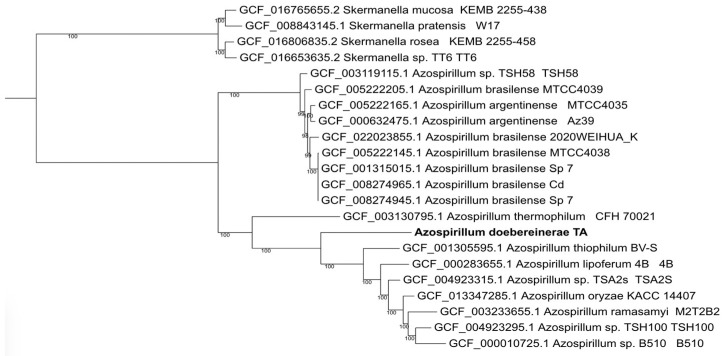
Phylogenomic tree of all available complete *Azospirillum* genomes. A total of 124 single-copy orthologs were obtained using BUSCO5, aligned individually using MAFFT and concatenated into a super matrix. The maximum-likelihood tree was constructed based on the Tamura–Nei model using IQ-TREE 2, with 1000 ultrafast bootstrap replications.

A phylogenomic analysis using 124 single-copy orthologs (dataset bacteria_odb10 from the OrthoDB database) showed that *Azospirillum* species form two major clades (Figure 2), one of which is a complex of nearly identical species—*A. argentinense* and *A. brasiliense*.

### 3.4. Functional Annotation

The functional classification of predicted genes of nitrogen metabolism in the TA genome was performed using Prokka and, additionally, via the eggnog-mapper pipeline. We explored the obtained results using the *Azospirillum brasilense* Sp7^T^ reference in the KEGG Pathway database. Nitrogen metabolism is very important for bacterial–plant interactions, and we identified the genes responsible for dissimilatory (*Nap*AB, *Nir*BD) and assimilatory (*Nas*AB) nitrate reductions, as far as denitrification (NirK, NirS) were detected. In addition, genes for the nitric oxide reduction (*nor*B, *no*rC) were identified, which make the *Azospirillum* TA strain unique by providing nitrogen availability for plants, thus contributing to its role as a PGPB.

A full set of genes including dinitrogen fixation structural genes (*ni*fDKH) and key genes for ammonium assimilation and the regulation of these processes were found (Table 3).

## 4. Discussion

For the first time, the complete genome sequence of a new strain of the nitrogen-fixing bacterium *Azospirillum doebereinerae* TA was obtained. It was isolated during a study in the Chenevaya taiga of Western Siberia, a unique fertile forest ecosystem known for its diverse plant growth-promoting bacteria (PGPB), and sequenced using the Illumina and Nanopore platforms in order to obtain complete high-quality genome assemblies.

The complete genome sequencing of the TA strain provided important information about its genetic composition and possible functional properties. This strain has interesting genetic characteristics, including several rRNA operons on different replicons, or “chromids” [15].

The strain’s placement within the *Azospirillum* genus was confirmed by the phylogenomic study, which also served as a foundation for comparative genomic research. According to the digital DNA-DNA hybridization (dDDH) and average nucleotide identity (ANI) values between strain TA and other related strains, the whole-genome evolution tree of strain TA and 17 other *Azospirillum* strains showed that it was a novel strain of *A. doebereinerae* (see Figure 2, Table 2).

The global nitrogen budget is largely controlled by microbiological processes, with nitrogen fixation being the main source of fixed nitrogen and denitrification acting as the largest sink. This prompted us to pay special attention to the genetic components of the strain TA genome involved in nitrogen fixing and other nitrogen metabolism processes. The identification of nitrogen fixation-related genes highlights the bacterium’s potential as a biofertilizer, improving soil fertility and promoting sustainable agricultural methods.

Nitrogen fixation was the first mechanism demonstrated to positively influence plant growth by *Azospirillum*, and much research has been conducted on this subject, with substantial information published (see, e.g., [25,26]). Long-term studies have shown that the beneficial effect is also associated with the synthesis of multiple phytohormones and plant regulators, phosphate solubilization, root system proliferation, enhanced water and mineral uptake, mitigation of environmental stressors, and competition with pathogens. The contribution of a single mechanism or the combination of several or multiple mechanisms may be involved in different situations. Recent studies have demonstrated the beneficial effect of *Azospirillum* inoculation in improving fertilizer efficiency and reducing application rates while maintaining crop yield [27].

A set of key genes encoding enzymes involved in nitrogen fixation was found in the genomic analysis of *A. doebereinerae* TA. In all known *Azospirillum*, the *nif*HDK genes encode, respectively, a nitrogenase iron (Fe) protein, a nitrogenase molybdenum-iron (MoFe) protein alpha chain, and a nitrogenase MoFe protein beta chain. The FeMo co-factor (FeMoCo) present in the MoFe protein binds N_2_, while the Fe protein uses the energy from ATP hydrolysis to drive the reduction of N_2_ to NH_4_^+^ by FeMoCo [28]. We used the presence of the catalytic *nif*HDK genes in the genome to predict the nitrogen fixation capacity of the TA strain and found all these genes. It is interesting to note that two copies of *nif*K and *nif*D were found. The presence of more than one *nif*HDK may increase nitrogen fixation; however, the selective advantage of such increased activity remains unclear.

Assimilatory and dissimilatory nitrate reduction processes represent the two ways available for nitrate reduction procedures. Assimilatory nitrate reduction occurs when an organism uses nitrogen from the environment as nitrate and absorbs it or integrates it into its own cell as an amino group. The nitrite reductase and nitrate reductase enzymes are encoded by the genes of the nitrite–nitrate (*NasAB*) assimilatory operon [29], which we discovered in the genome sequence of strain TA.

Because nitrate is used as the terminal electron acceptor and the process is carried out as energy production (ATP synthesis), the dissimilatory reduction of nitrate is also known as nitrate respiration. This process has two types: the first forms ammonium (DNRA), while the second produces gaseous nitrogen compounds (denitrification) [30]. In the *A. doebereinerae* TA strain, the *NirBD* operon was found to encode a cytochrome C nitrite reductase that reduces nitrite to ammonium, and the *NapAP* operon was found to encode a periplasmic nitrate reductase that reduces nitrate to nitrite. Considering that dissimilatory nitrate reduction is a poorly understood ecological mechanism, this is an extremely intriguing discovery. It has been suggested that this is not essential for the nitrogen cycle, occurs in a limited number of anaerobic conditions (sewage sludge, marine sediments), and is mediated by a small number of microorganisms [31], while the involvement of *Azospirillum* in this process is unknown. However, under certain situations, DNRA might be essential to microorganisms or plants that prefer ammonium to nitrate for absorption. The cost of NH_4_^+^ absorption and assimilation is lower than that of NO_3_^−^; therefore, plants may prefer ammonium [32]. DNRA keeps nitrogen in the soil and does not aid in its removal, in contrast to denitrification. When the C-to-NO_3_^−^ ratio is large, it is preferred over denitrification and operates in low-NO_3_^−^ circumstances with high carbon (C) availability [33]. While denitrification results in gaseous losses in the form of nitrogen gas or the greenhouse gas nitrous oxide (N_2_O), DNRA conserves nitrogen. Therefore, it would be advantageous to encourage *Azospirillum* bacteria in agricultural soils to support nitrogen status, in addition to their PGPB activity.

In order to obtain energy, bacteria also use a biological respiratory process called denitrification to convert nitrate (NO_3_^−^) and/or nitrite (NO_2_^−)^ into gaseous products (NO, N_2_O, or N_2_) [29]. The genome of strain TA contains the main functional genes associated with denitrification: *nar, nir*, and *nor.* We identified *NarAP* genes, which catalyze the initial stage of denitrification and are mediated by respiratory membrane-bound nitrate reductases. The reduction of nitrite to the nitric oxide, a crucial step in the denitrification pathway, is catalyzed by cytochrome cd1-containing nitrite reductases, encoded by the *nirS* gene, and copper-dependent nitrite reductase, encoded by the *nirK* gene. These genes have been used as functional markers for denitrification in environmental surveys. In the genome of the TA strain, we found both genes *NirK* and *NirS*. Nitric oxide reductase catalyzes the third phase, which is the reduction of NO to N_2_O.

The TA genome also contains the *NorBC* operon. Since the nos genes determining the final step of denitrification were not detected, the final gaseous product produced by the *A. doebereiner* TA strain during denitrification is N_2_O. Interestingly, most *Azospirillum* strains contain only the *NirK* or *NirS* genes. As previously found in the genome of the *A. brasilense* strain Sp7^T^, which we used as a reference in the functional annotation, both genes were found in the genome of the *A. doebereinerae* TA strain [29].

## 5. Conclusions

We expect that the availability of the complete genome sequence of *A. doebereinerae* AT strain will contribute to a new understanding of plant growth promotion and soil fertility maintenance mechanisms through genomic comparisons between available complete genomes of *Azospirillum* strains. Further studies are needed to explore the full metabolic capabilities and potential applications of this new strain.

## Figures and Tables

**Table 1:** Genome statistics of *Azospirillum doebereinerae* TA.

Replicon	1	2	3	4	5	6	7	All
Size, bp	2,835,377	1,157,394	1,143,355	891,684	656,579	200,787	52,165	6,937,341
GC content	68.83	68.89	69.40	68.80	68.84	66.91	68.93	68.66
Coverage	68	64	63	64	63	103	52	-
Mult.	1	1	1	1	1	2	1	1
16S rRNA	3	3	1	1	0	0	0	8
5S rRNA	3	3	1	1	0	0	0	8
23S rRNA	3	3	1	1	0	0	0	8
tRNA	55	14	5	8	2	1	0	85
All CDs	2690	998	1022	758	563	161	56	6248
CDs with predicted function	1388	527	545	372	267	70	4	3173
Mobilome	16	7	7	29	6	4	0	69
CRISPR	2	0	0	1	0	0	0	3

**Table 2:** Comparative genomic analysis of *Azospirillum* TA with *Azospirillum* genomes.

Genome	ANIb [%] ^1^	ANIm [%] ^2^	Z-Score	Size (bp)	GC%
*Azopirillum* TA	100	100	1	6,916,495	68.8
*Azospirillum doebereinerae* BF-21-2S	99.61	99.70		6,880,504	68.9
*Azospirillum doebereinerae* GSF71 ^T^	98.68	98.95	0.99636	7,000,062	68.88
*Azospirillum thiophilum* DSM 21654	81.48	86.59	0.9644	7,637,524	68.15
*Azospirillum palustre* B2	81.37	86.49	0.95698	7,997,491	67.80
*Azospirillum melinis* TMCY0552	81.23	86.41	0.95589	7,970,174	67.70
*Azospirillum oryzae* COC8	80.73	86.35	0.95548	6,755,201	67.36
*Azospirillum lipoferum* 59b	80.71	86.24	0.94835	7,987,183	67.26
*Azospirillum* sp. B510	80.65	86.44	0.9539	7,599,738	67.61
*Azospirillum griseum* L-25-5 w-1	79.62	85.94	0.89421	5,951,384	66.57
*Azospirillum argentinense* Az39	78.64	85.60	0.97928	7,391,279	68.56
*Azospirillum rugosum* IMMIB AFH-6	78.56	85.46	0.96455	7,798,764	68.86
*Azospirillum baldaniorum* Sp245	78.53	85.60	0.97853	7,530,241	68.44
*Azospirillum formosense* CC-NFb-7	78.22	85.54	0.97697	6,161,078	68.63
*Azospirillum tabaci* W712	78.21	85.51	0.97492	6,322,916	68.66
*Azospirillum brasilense* Sp 7	78.18	85.50	0.97781	6,587,527	68.33
*Azospirillum halopraeferens* DSM 3675	75.10	84.08	0.94308	6,508,482	70.71

1. Average nucleotide identity (ANI) between genomes based on BLAST algorithm. 2. ANI based on the MUMmer algorithm.

**Table 3:** *Azospirillum doebereinerae* strain TA genes involved in nitrogen fixation, nitrogen assimilation, and regulation of these processes.

Genes	EC Number	Product
*nif*H	1.18.6.1	Structural gene dinitrogenase reductase (Fe protein)
*nifD_1, nifD_2*	1.18.6.1	Structural gene dinitrogenase (MoFe protein, α-subunit)
*nifK_1, nifK_2*	1.18.6.1	Structural gene dinitrogenase (MoFe protein, β-subunit)
*nifA*	-	Transcriptional activator of the nitrogen fixation (*nif*) genes
*nifW*	-	Nitrogenase-stabilizing/protective protein
*amtB_1, amtB_2*	-	Structural gene ammonium transporter
*glnB*	-	N-signal transmitter protein
*ntrB*	-	Sensor protein of two-component regulatory system, involved in general nitrogen control
*ntrC*	-	Sensor protein of two-component regulatory system, involved in general nitrogen control
*draT*	2.4.2.37	Dinitrogenase reductase ADP ribosyl-transferase
*draG*	3.2.2.24	Dinitrogenase reductase activating glucohydrolase

## Data Availability

The complete genome assembly was deposited in the NCBI GenBank (BioProject PRJNA1182660). The 16S rRNA gene sequence was also deposited in the NCBI GenBank, although separately, under accession number ON_103335.1.
